# Cardiac operation under cardiopulmonary bypass during pregnancy

**DOI:** 10.1186/s13019-020-01136-9

**Published:** 2020-05-13

**Authors:** Yanli Liu, Fengzhen Han, Jian Zhuang, Xiaoqing Liu, Jimei Chen, Huanlei Huang, Sheng Wang, Chengbin Zhou

**Affiliations:** 1 Department of Obstetrics, Guangdong Provincial People’s Hospital, Guangdong Academy of Medical Sciences, No. 106 Zhongshan 2nd road, Guangzhou, 510080 Guangdong China; 2 Department of Cardiovascular Surgery, Guangdong Cardiovascular Institute, Guangdong Provincial Key Laboratory of South China Structural Heart Disease, Guangdong Provincial People’s Hospital, Guangdong Academy of Medical Sciences, Guangzhou, Guangdong China; 3 Department of Epidemiology, Guangdong Cardiovascular Institute, Guangdong Provincial Key Laboratory of South China Structural Heart Disease, Guangdong Provincial People’s Hospital, Guangdong Academy of Medical Sciences, Guangzhou, Guangdong China; 4 Department of Anesthesia, Guangdong Provincial People’s Hospital, Guangdong Academy of Medical Sciences, Guangzhou, Guangdong China

**Keywords:** Cardiac operation, Cardiopulmonary bypass, Pregnancy, Outcome

## Abstract

**Background:**

Certain pregnant women suffer from cardiac pathology,and a few of them need cardiac operations under cardiopulmonary bypass during pregnancy. Feto-neonatal and maternal outcomes have not been sufficiently described.

**Methods:**

We conducted a retrospective review of 22 cases of women undergoing cardiac operations under cardiopulmonary bypass during pregnancy in our hospital from Jan.2014 to Mar.2019.

**Results:**

All 22 patients were alive after treatment. The types of cardiac disorders included congenital heart defects, rheumatic heart disease,infective endocarditis,aortic dissection, obstruction and/or thrombosis of a prosthetic valve. Only one case was a twin pregnancy,and the other 21 cases were singletons. Four fetuses died in the utero after surgery. Three patients chose termination of the pregnancy after the cardiac operations: one fetus was detected abnormity of the brain and the other two patients abandoned pregnancy. Fourteen fetuses were alive and born without any abnormity. Two fetuses suffered from neonatal intracranial hemorrhage and died after birth.

**Conclusions:**

Cardiac operation under cardiopulmonary bypass during pregnancy is a challenge for physicians in multidisciplinary teams. Strictly evaluating the indication is vital. On the other hand, some patients can benefit from this management.

## Background

Heart disease complicates more than 1% of pregnancies and is now the leading cause of indirect maternal deaths [1]. Pregnancy creates a great burden on the cardiovascular system and can result in decompensation in women with underlying cardiac disease. To minimize the maternal and fetal risks, the first choice of treatment should be medical. In cases that are refractory to medical treatment, however, corrective cardiac operations should be undertaken [2]. As the Guangdong provincial obstetrical cardiology intensive care center in China, our hospital has accumulated a significant amount of clinical data of pregnant women with heart disease receiving cardiac operations under cardiopulmonary bypass during pregnancy. To investigate feto-neonatal and maternal outcomes, we conducted this study.

## Materials and methods

### Subject

We searched in our medical record database from Jan.2014 to Mar.2019. The search terms included “pregnancy”, “cardiopulmonary bypass” and “cardiac operation”. We obtained 22 copies of the patients’ medical materials containing the entire pregnancy course and fetal outcomes with their consent.

### NYHA classes

The NYHA classification was developed in 1928 to describe an overall cardiac appraisal of the status of a patient with heart disease. It was divided into four classes [3]: Class I: Patients with cardiac disease but without resulting limitation of physical activity. Ordinary physical activity does not cause undue fatigue, palpitation, dyspnea, or anginal pain. Class II: Patients with cardiac disease resulting in a slight limitation of physical activity. They are comfortable at rest. Ordinary physical activity results in fatigue, palpitation, dyspnea, or anginal pain. Class III: Patients with cardiac disease resulting in marked limitation of physical activity. They are comfortable at rest. Less than ordinary activity causes fatigue, palpitation, dyspnea, or anginal pain. Class IV:Patients with cardiac disease resulting in inability to carry on any physical activity without discomfort. Symptoms of heart failure or anginal syndrome may be present even at rest. If any physical activity is undertaken, discomfort increases.

### Cardioplegia technique

Adequate myocardial protection is essential for achieving successful outcomes of any surgical procedure necessitating cardiac arrest. The Del Nido solution (blood and crystalloid mixed formula) was used in all the cardiac operations of our study. The route of administration was antegrade or combined antegrade & retrograde.

### Cardiac surgical procedures

Corrective cardiac operations consisted of mitral or/and tricuspid valve repair, aortic valve replacement (AVR),mitral valve replacement (MVR), ruptured sinus of Valsalva repair, atrial septal defect closure, ventricular septal defect closure, right ventricle outlet obstruction repair, prosthetic mitral/aortic valve thrombectomy and Betall procedure.

### Maternal, fetal and neonatal complications after operation

The most common maternal complication was arrhythmia after operation. Fetal and neonatal complications included stillbirth, preterm delivery (< 37 weeks of gestation), neonatal intracranial hemorrhage and death.

### Statistical analysis

A retrospective analysis was performed. Measurement data and enumeration data were expressed as mean ± standard deviation (SD) or frequencies.

## Results

### Patient general information

The average age of the patients was 29.5 ± 5.4 years, with an age range of 21 to 42 years. Half the patients were nulliparous (*n* = 12, 54.5%). There was one twin pregnancy(*n* = 1, 4.5%)and the other patients were singletons(*n* = 21, 95.5%). The patient’s characteristics are listed in Table 1.

**Table 1:** Patient’s characteristics

Patient No.	Age (y)	Gravidity	Parity	Singleton /twin (S/T)	Weight during operation (kg)	Type of heart disease	NYHA functional classification	Weeks of gestation during operation (w)
1	32	1	0	S	43	ASD (PAH accompanied)	II	22^+ 4^
2	36	4	1	S	60	MR (PAH accompanied)	II	20^+ 4^
3	33	3	0	T	63	DCRV	II	26^+ 5^
4	35	5	2	S	60	MS (PAH accompanied)	III	18^+ 6^
5	25	1	0	S	49	Prosthetic AV stuck	III	20^+ 6^
6	42	3	1	S	72	MS (PAH accompanied)	IV	27^+ 3^
7	30	2	1	S	66	MS + ASD (PAH accompanied)	II	23^+ 4^
8	23	1	0	S	48	AR	II	18^+ 1^
9	29	4	2	S	49	IE + MR	IV	25^+ 5^
10	24	2	0	S	41	ASD (PAH accompanied)	II	20^+ 4^
11	26	1	0	S	49	Prosthetic AV stuck	IV	19^+ 5^
12	28	3	1	S	51	VSD (PAH accompanied)	II	24^+ 2^
13	25	4	1	S	55	ASD (PAH accompanied)	II	22^+ 3^
14	28	5	0	S	57	Prosthetic AS	II	30^+ 5^
15	37	2	1	S	74	VSD + AR	II	20^+ 3^
16	28	3	0	S	47	ASD (PAH accompanied)	II	25^+ 3^
17	36	3	1	S	50	AD (Stanford type A)	III	23^+ 6^
18	26	2	1	S	68	IE	III	26
19	30	1	0	S	49	MS (PAH accompanied)	III	28
20	24	1	0	S	45	ASD + VSD (PAH accompanied)	III	25^+ 6^
21	21	1	0	S	48	Ruptured sinus of Valsalva of the right coronary cusp+IE	IV	21
22	25	1	0	S	48	Prosthetic AS	III	26^+ 4^

*y* Year, *kg* Kilogram, *w* Week, *S* Singleton, *T* Twin, *ASD* Atrial septal defect, *VSD* Ventricular septal defect, *MR* Mitral valve regurgitation, *DCRV* Double cavity of right ventricle, *MS* Mitral valve stenosis, *AR* Aortic valve regurgitation, *IE* Infective endocarditis, *MR* Mitral valve regurgitation, *PAH* Pulmonary artery hypertension, *AD* Aortic dissection, *AS* Aortic valve stenosis

### Cardiac surgical procedure, intraoperatory parameters and fetal outcomes

There were 22 patients with different types of heart diseases who received cardiac operations under cardiopulmonary bypass during pregnancy. The composition and proportion distribution of these patients by the type of heart disease,weeks of gestation during operation, NYHA functional classification, cardiac surgical procedure, intraoperatory parameters and fetal outcomes are presented in Table 1,Table 2 and Table 3.

**Table 2:** Cardiac surgical procedure, intraoperatory parameters and fetal outcomes

Patient No.	Cardiac surgical procedure	Size of the cardiac valves/defects (mm)	Aortic cross-clamp time (minutes)	CPB time (minutes)	CPB maximum flow (L)	CPB minimum temperature (°C)	Fetal outcomes
1	atrial septal defect closure	45	10	40	3.7	35.8	term birth, alive
2	mitral and tricuspid valve repair	/	60	96	5.8	35.3	term birth, alive
3	right ventricle outlet obstructio-n repair	/	20	47	4.7	35.4	preterm birth, alive
4	MVR	27	47	75	4.5	36	termination of pregnancy
5	MVR	25	66	102	5.5	35.5	term birth, alive
6	MVR	25	31	52	5	36.5	preterm birth, alive
7	MVR+ atrial septal defect closure	27/13	35	62	4.5	35.7	preterm birth, alive
8	AVR	24	75	112	4.6	34.4	term birth, alive
9	prosthetic mitral valve thrombect-omy + mitr-al valve repair	/	101	133	4.9	34.8	abnormity of the brain, termination of pregnancy
10	atrial septal defect closure	35	18	35	3.5	34.9	term birth, alive
11	MVR	23	120	170	4.8	30	term birth, alive
12	ventricula-r septal defect closure	13.8	30	72	4.5	35.1	term birth, alive
13	atrial septal defect closure	21.7	21	40	4.2	35.8	term birth, alive
14	AVR	19	95	122	5	34.7	preterm birth,death
15	ventricula-r septal defect closure+ AVR	16.3/23	78	97	5.2	36.6	term birth, alive
16	atrial septal defect closure	30	13	25	4.5	36.2	term birth, alive
17	Betall procedure	/	172	241	4.5	30	death in utero
18	MVR	29	32	57	4.4	36.3	death in utero
19	MVR	25	31	52	4.98	36	preterm birth, death
20	atrial septal defect closure+ ventricula-r septal defect closure	12/25	35	74	4.3	34.1	death in utero
21	ruptured sinus of Valsalva repair+ valves thrombect-omy	/	163	211	4.1	33.1	termination of pregnancy
22	prosthetic aortic valve thrombect-omy	/	65	174	4.0	17.7	death in utero

*mm* Millimetre, *L* Litre, *CPB* Cardiopulmonary bypass, *AVR* Aortic valve replacement, *MVR* Mitral valve replacement

**Table 3:** Summary of indications for cardiac operation

Indication	n(%)
Congenital heart defect	8(36.4%)
Rheumatic heart disease	7 (31.8%)
Infective endocarditis	2 (9.1%)
Aortic dissection	1 (4.5%)
Obstruction and thrombosis of prosthetic valve	4 (18.2%)

### Feto-neonatal and maternal outcomes

All 22 patients were alive after treatment. Three cases were complicated by arrhythmia after operations, especially atrial fibrillation, which needed medications. Four fetuses died in the utero after operations. Three patients chose termination of the pregnancy: one fetus was detected a brain abnormity and the other two patients abandoned pregnancy. Fourteen fetuses were alive and born without any abnormity. Two fetuses had complicated neonatal intracranial hemorrhage and died after birth. Feto-neonatal outcomes and mode of delivery are presented in Table 4.

**Table 4:** Feto-neonatal outcomes and mode of delivery

Mode of delivery	n(%)	Feto-neonatal outcome
Cesarean section	14 (63.6%)	14 fetuses were alive without any abnormity(9 fetuses were term deliveries, and the other 5 were preterm deliveries). One fetus manifested intracranial hemorrhage at 36 weeks of gestation and died after birth.
Induced labor (vaginal delivery)	1 (4.5%)	Neonatal intracranial hemorrhage and died after birth
Spontaneous abortion	4 (18.2%)	Intrauterine death after operation
Termination of pregnancy	3 (13.6%)	1 fetus was detected abnormity of the brain. 2 patients abandoned pregnancy

## Discussion

Heart disease is the primary cause of maternal and fetal death in 1–4% of pregnancies. Pregnancy creates an increased burden on the maternal cardiovascular system and can result in decompensation in women with underlying cardiac disease. To minimize the maternal and fetal risks, the first choice of treatment should be medical. However, in some cases, medical therapy is not always sufficient,and open heart operation might be necessary [4]. In 1958, Leyse and colleagues [5] first used cardiopulmonary bypass (CPB) in a heart operation during pregnancy. After the initial trials, pregnant women have been recognized to tolerate CPB as well as non-pregnant women, but the effects of CPB on the fetus have varied [6]. Several review articles, reported the maternal mortality rate ranged from 1.5 to 5%, and the fetal mortality rate has ranged from 16 to 33% [4, 6]. Currently, reported maternal mortality for cardiac operations is similar to the mortality rate for non-pregnant female patients [7]. Therefore, CPB during pregnancy has a greater effect on the fetus than mother. In our report, the maternal mortality rate was 0%,and fetal mortality rate was 18.2%, as same as the above mentioned reviews.

The present study demonstrated that mitral and/or aortic valve disorders were the most common surgical indications for CPB during pregnancy, although it has been recognized that coronary arterial disease is increasingly prevalent in gynecological patients [8]. The latter, however, could be managed interventionally in most patients, avoiding the risk associated with CPB for feto-neo-natal outcomes. In our report, the indications for surgical procedure under CPB during pregnancy consisted of congenital heart defect (ASD, DCRV, VSD), rheumatic heart disease (mitral or aortic valve disorders),infective endocarditis,aortic dissection, obstruction and thrombosis of the prosthetic valve. Seven patients (all with a congenital heart defect)accompanied moderate to severe PAH, which could result in sudden death and greatly increase the maternal and fetal risk. Consequently, we performed cardiac operations during pregnancy to maintain the pregnancy and to decrease the risk of adverse feto-neonatal outcomes. Other indications were life-threatening diseases, such as severe MS/AR, infective endocarditis,aortic dissection (Stanford type A), obstruction and thrombosis of the prosthetic valve. All patients were alive,and 3 cases had complicated arrhythmia after operations, especially atrial fibrillation. There were no other complications. The results indicate that cardiac operations can be performed during pregnancy with remarkable safety for mothers.

Pregnant women who have cardiac operations requiring CPB must face a nonphysiologic hemodynamic status where the tolerance is not clearly known, which can adversely affect the fetus [4]. CPB can compromise utero-placental perfusion and fetal development by potential adverse effects such as coagulation and blood component alterations, the release of vasoactive substances from leukocytes, complement activation, particulate and air embolism, nonpulsatile flow, hypothermia and hypotension [2].Three main pathophysiological changes can occur in pregnant patients under CPB: uterine contraction, placental hypo-perfusion and fetal hypoxia. Utero-placental hypo-perfusion and fetal hypoxia subjected to sustained uterine contractions during CPB are considered risk factors for fetal death [9]. Despite the limited experimental data regarding the effect of CPB on uterine/placental blood flow and its effect on the fetus, it has been postulated that pulsatile, high-flow, high-pressure, normothermic bypass poses the least risk to the fetus [10, 11].According to the above theories we applied high-flow, high-pressure, normothermic bypass to the patients and shortened the operation time to greatly decrease the influence on the fetus. Finally,the fetuses gained good outcomes,and the mortality rate was 18.2%, lower than that reported in recent literature. Fourteen fetuses were alive and born without any abnormity. Unfortunately, two fetuses suffered neonatal intracranial hemorrhage and died after birth. However, we do not think it was associated with the operation or the CPB during pregnancy. The inappropriate use of Warfarin after operations was the main cause. The results indicate that cardiac operations can be performed during pregnancy with a degree of safety for fetus.

## Conclusion

In conclusion, the decision to subject a pregnant woman to operation must be made by a team composed of an obstetrician, a cardiologist, an anesthesiologist and a neonatologist. Cardiac operation under CPB during pregnancy is a challenge for physicians in multidisciplinary teams. Strictly evaluating the indication is vital. On the other hand, some patients can benefit from this form of case management.

## Data Availability

The data were presented in the main manuscript.

## Abbreviations

y: Year
kg: Kilogram
w: Week
S: Singleton
T: Twin
mm: Millimetre
L: Litre
ASD: Atrial septal defect
VSD: Ventricular septal defect
MR: Mitral valve regurgitation
DCRV: Double cavity of right ventricle
MS: Mitral valve stenosis
AR: Aortic valve regurgitation
IE: Infective endocarditis
MR: Mitral valve regurgitation
PAH: Pulmonary artery hypertension
AD: Aortic dissection
AS: Aortic valve stenosis
AVR: Aortic valve replacement
MVR: Mitral valve replacement
SD: Standard deviation
CPB: Cardiopulmonary bypass

## References

[1] Simpson LL (2012). Maternal cardiac disease: update for the clinician. Obstet Gynecol.

[2] Chambers CE, Clark SL (1994). Cardiac surgery during pregnancy. Clin Obstet Gynecol.

[3] Hurst JW (2006). The value of using the entire New York heart Association’s classification of heart and vascular disease. Clin Cardiol.

[4] Mahli A, Izdes S, Coskun D (2000). Cardiac operations during pregnancy: review of factors influencing fetal outcome. Ann Thorac Surg.

[5] Leyse R, Ofstun M, Dillard DH, Merendino KA (1961). Congenital aortic stenosis in pregnancy, corrected by extracorporeal circulation: offering a viable male infant at term but with anomalies eventuating in this death at four months of age—report of case. JAMA.

[6] Becker RM (1983). Intracardiac surgery in pregnant women. Ann Thorac Surg.

[7] Gopal K, Hudson IM, Ludmir J, Braffman MN, Ewing S, Bavaria JE, Wong KL, Bridges CR (2002). Homograft aortic root replacement during pregnancy. Ann Thorac Surg.

[8] Jantzen J-P, Bader W (2011). The cardiac risk patient presenting for gynecological surgery. Geburtshilfe Frauenheilkd.

[9] Yuan SM (2014). Indications for cardiopulmonary bypass during pregnancy and impact on fetal outcomes. Geburtshilfe Frauenheilkd.

[10] Westaby S, Parry AJ, Forfar JC (1992). Reoperation for prosthetic valve endocarditis in the third trimester of pregnancy. Ann Thorac Surg.

[11] Pomini F, Mercogliano D, Cavalletti C, Caruso A, Pomini P (1996). Cardiopulmonary bypass in pregnancy. Ann Thorac Surg.

